# “We can’t carry the weight of the whole world*”*: illness experiences among Peruvian older adults with symptoms of depression and anxiety

**DOI:** 10.1186/s13033-020-00381-8

**Published:** 2020-07-10

**Authors:** Oscar Flores-Flores, Alejandro Zevallos-Morales, Ivonne Carrión, Dalia Pawer, Lorena Rey, W. Checkley, J. R. Hurst, T. Siddharthan, Jose F. Parodi, Joseph J. Gallo, Suzanne L. Pollard

**Affiliations:** 1 Facultad de Medicina Humana, Centro de Investigación del Envejecimiento (CIEN), Universidad de San Martin de Porres, Lima, Peru; 2 Asociación Benéfica PRISMA, Lima, Peru; 3 Facultad de Medicina Humana, Universidad de San Martin de Porres, Lima, Peru; 4 Pontificia Universidad Católica del Perú, Lima, Peru; 5 Division of Pulmonary and Critical Care, School of Medicine, Johns Hopkins University, Baltimore, MD USA; 6 Department of International Health, Bloomberg School of Public Health, Johns Hopkins University, Baltimore, MD USA; 7 Center for Global Non-Communicable Disease Research and Training, School of Medicine, Johns Hopkins University, Baltimore, MD USA; 8 UCL Respiratory, University College London, London, UK; 9 Department of Mental Health, Bloomberg School of Public Health, Baltimore, MD USA; 10 Department of General Internal Medicine, School of Medicine, Johns Hopkins University, Baltimore, MD USA

**Keywords:** Qualitative research, Anxiety, Depression, Aging

## Abstract

**Background:**

Despite the high levels of depression and anxiety symptoms in old age, the use of mental health services in this population is low. Help-seeking behaviors are shaped by how an individual perceives and experiences their illness. The objective of this study was to characterize the illness experiences of Peruvian older adults with depression and anxiety symptoms in order to lay the foundation for tailored community-based mental health interventions.

**Methods:**

In this qualitative study, we conducted in-depth interviews with a purposively selected sample of older adults (≥ 60 years) from peri-urban areas of Lima, Peru. We included individuals with only depressive symptoms (Patient Health Questionnaire-9 ≥ 10), only anxiety symptoms (Beck Anxiety Inventory ≥ 16), with depressive and anxiety symptoms, and older adults who mentioned they had received mental health treatment/care. The interview guide included the following topics: perceptions and experiences about depression and anxiety; perceptions about the relationship between physical chronic diseases and mental health; experiences with mental health professionals and treatments, and coping mechanisms. Data collection was conducted between October 2018 and February 2019.

**Results:**

We interviewed 38 participants (23 women, 15 men) with a mean age of 67.9 years. Participants’ ideas and perceptions of depression and anxiety showed considerable overlap. Participants attributed depression and anxiety mainly to familial and financial problems, loneliness, loss of independence and past traumatic experiences. Coping strategies used by older adults included ‘self-reflection and adaptation’ to circumstances, ‘do your part’, and seeking ‘emotional support’ mainly from non-professionals (relatives, friends, acquaintances, and religion).

**Conclusions:**

Illness experiences of depression and anxiety set the pathway for tailored community-based mental health interventions for older adults. Overlapping narratives and perceptions of depression and anxiety suggest that these conditions should be addressed together. Mental health interventions should incorporate addressing areas related to depression and anxiety such as prevention of loss of independence, trauma, and loneliness. Good acceptability of receiving emotional support for non-professionals might offer an opportunity to incorporate them when delivering mental health care to older adults.

## Background

Two-thirds of the world’s older adults live in low and middle income countries (LMICs), where the population 60 years of age and older is growing at a faster rate than in high-income countries [1]. Societies and health systems in LMICs face challenges to adapting and promoting wellbeing in aging populations. Mental health disorders, such as depression and anxiety, are associated with poor quality of life in older adults; yet, these two conditions are often neglected, under-diagnosed, and inadequately treated [2–4]. In Peru, 85% of adults requiring mental health care do not receive it [5]. A multi-center study found that up to 23% of older adults living in urban areas of Peru have significant depressive symptoms, and 10% have symptoms of anxiety [6, 7]. Given the high prevalence of depression and anxiety symptoms and low use of mental health services, development of appropriate, community-based, scalable strategies to provide quality care for older people are critical for promoting the wellbeing of older populations [8].

Studies of the prevalence of depression and anxiety in older adults do not offer insight into the explanatory models of illness (i.e., how they describe their experience, causes of depression and anxiety, and what to do about it) [9]. Interventions that take into account one’s understanding of illness could lead to improved engagement and better health outcomes [10]. Approaches with a narrow focus or use of a bio-medical model may overlook important social, contextual, and cultural factors [11]. In 2012, the Peruvian government approved a series of actions to reform its mental health services, including bringing services to the community level [5, 12]; thus, studies focused on community perspectives are timely.

The purpose of our study was to characterize the lived experiences of depression and anxiety of older adults in peri-urban areas of Lima, Peru. Because depression and anxiety frequently co-exist and may not be clearly differentiated by general public [13], we considered both. Few studies have explored the perceptions of depression or anxiety in older adults from Latin America [14, 15]. In three recent systematic reviews of qualitative studies about depression in older adults, none included populations from Latin America [16–18]. We chose a qualitative approach, using open-ended questions to elicit the older person’s point of view regarding depression and anxiety. Our ultimate goal was to lay the foundation for mental health interventions tailored to the needs of older adults from low resource settings in Peru and similar contexts.

## Methods

### The parent study: GECO

The present study was nested within a community-based parent project called the Global Excellence for Chronic Obstructive Pulmonary Disease Outcomes (GECo) Study [19]. The sample was obtained from two peri-urban districts of Lima, Peru: San Juan de Miraflores (SJM) and Villa El Salvador (VES).

### Sample selection

GECo study included a random sample of the 3500 individuals drawn from the general population to screen for chronic obstructive pulmonary disease (COPD). During the initial visit, individuals were screened for COPD, and simultaneously, trained fieldworkers administered several health questionnaires, included the Spanish validated versions of the Patient Health Questionnaire-9 (PHQ-9) and the Beck Anxiety Inventory (BAI) to assess depression and anxiety symptoms [20, 21]. Individuals with active pulmonary tuberculosis, or who were unable to perform spirometry due to severe cognitive or physical impairment, or had myocardial infarction or had eye, thoracic, or abdominal surgery in the previous 3 months, were excluded.

For the present qualitative study, we purposively selected older adults (> 60 years-old) who had moderate-severe depressive symptoms (PHQ-9 ≥ 10), moderate-severe anxiety symptoms (BAI ≥ 16), and moderate-severe symptoms of both (PHQ-9 ≥ 10 and BAI ≥ 16), as well as older adults that did not reach the threshold score for BAI nor PHQ-9 (low depressive/anxiety symptoms) but who during GECo visits mentioned that they had received some form of mental health care (anxiolytics, antidepressants, visit with psychologist or psychiatrist).

We approached older adults by telephone and in person to carry out informed consent, and asked if they would participate in an in-depth interview in their homes regarding their mental and physical health. Interviews were conducted in the local language (Spanish). Two interviewers attended each interview; one led the interview and another took notes. We asked permission to digitally record the interview. Interviews lasted between 40 and 70 min. During interviews, we discovered two older adults had been diagnosed with schizophrenia. One was excluded because he could not follow the interview coherently. The other older adult was stable, coherent, and had been on treatment for 30 years, and his interview was included.

### Interview guide

We initially developed the interview guide through extensive discussion among authors and based on previous studies/guides about depression in old age from other settings [22, 23]. The guide included the following topics: ideas and perceptions about depression and anxiety, including personal experiences; perceptions about the relationship between physical chronic diseases and mental health; experiences and perceptions about mental health professionals and treatments (pharmacologic and non-pharmacologic), and coping mechanisms. The initial interview guide was piloted with two older adults (> 60 years-old) from the study community. We found it difficult to obtain quality information when asking direct questions about depression and anxiety. Therefore, in subsequent interviews, we included two case-vignettes based on the Spectrum study [22] to more easily approach the topic of depression, and doing so ultimately provided richer information. The interview guide is provided in Additional file 1.

### Data analysis

Interviews were digitally recorded with consent and transcribed verbatim by external expert personnel. We (OFF, AZM, LR, IC) read the initial four transcripts and generated preliminary coding categories through an inductive process. To increase reliability of coding, every transcript was reviewed by at least two researchers [24]. Researchers (OFF, AZM, LR, IC, SP, JG) met to discuss the codes that were identified individually. We then used the constant comparative method to move iteratively between codes and texts to identify emerging themes [25–27] until consensus was reached. To manage the data, we used MAXQDA software (VERBI GmbH, Berlin, Germany, Version 18.2). The final quotes were translated by a bilingual author (SP) from Spanish to English.

### Ethical considerations

Ethical approval was obtained from the Institutional Review Boards at Johns Hopkins University and A.B. PRISMA. Researchers in the study completed a training course in ethics and human subject protections, certified by the National Institutes of Health (NIH).

## Results

### Sample characteristics

We invited 44 individuals to participate in this study. Nevertheless, five refused to participate due to lack of time or interest. One individual was excluded during the interview due to untreated schizophrenia. As a result, we had a sample of 38 participants (23 women and 15 men). Table 1 describes the social and clinical characteristics of the participants. In all, 31 participants were considered ‘younger old’ (60–74 years) and seven were ‘oldest old’ (≥ 75 years). One man lived alone, while the rest of the participants lived with one or more family members. Eleven people (28.9%) had only depressive symptoms, 11 people had only symptoms of anxiety, and 11 had symptoms of anxiety and depression. Fourteen (41.2%) reported having visited a psychologist or psychiatrist during their lifetime, and seven (21.9%) had taken medications related to depression or anxiety symptoms.

**Table 1:** Social and clinical characteristics of sample population (n = 38)

Characteristics	Total (n = 38)
Age in years (n = 38) (mean, s.d.)	67.95 ± 7.79
Sex (n = 38)	
Male	15 (39.5)
Female	23 (60.5)
Education (n = 38)	
University or College	2 (5.2)
High school (complete/incomplete)	20 (52.6)
Primary school (complete/incomplete)	14 (36.8)
None	2 (5.3)
Marital status (n = 32)	
Married	15 (46.9)
Separated, divorced or single	8 (25.0)
Widow	9 (28.1)
Living status (n = 37)	
Alone	1 (2.7)
With spouse and others	13 (35.2)
Without spouse but with others	23 (62.1)
Work status* (n = 34)	
Doing work that generates income	17 (50.0)
Not doing work that generates income	17 (50.0)
Multi-morbidities** (n = 38)	
None	7 (18.4)
1 or 2 diseases	24 (63.2)
3 or more diseases	7 (18.4)
Taking medications for anxiety or depressive symptoms (n = 32)	
Yes	7 (21.9)
No	25 (78.1)
Experience with psychology/psychiatry (n = 34)	
Yes	14 (41.2)
No	20 (58.8)
Anxiety and depression status (n = 38)	
Only depressive symptoms (PHQ-9 ≥ 10)	11 (28.9%)
Only anxiety symptoms (BAI ≥ 16)	11 (28.9%)
Depressive and anxiety symptoms	11 (28.9%)
No depressive or anxiety symptoms	5 (13.3%)
Health insurance (n = 38)	
Has insurance	9 (23.7)
No insurance	29 (76.3)

*Work status: We defined ‘work’ as any activity that serves as a source of income (e.g. selling handmade fabrics, carpenters, taxi-drivers); almost all who worked were working informally

**Multi-morbidities: self-reported hypertension, diabetes mellitus, asthma, arthritis, valvulopathies, schizophrenia, and bronchitis/COPD

**Fig. 1:**
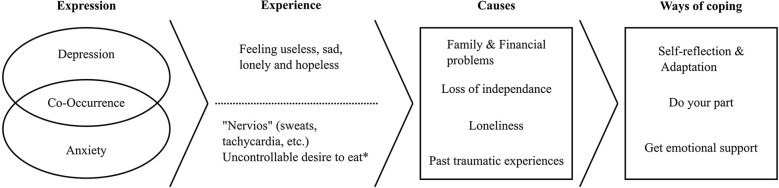
Summary of emerging themes regarding the illness experiences of depression and anxiety among Peruvian older adults. According to Peruvian older adults, depression was described mainly as feeling sad, useless, lonely, and hopeless. Anxiety was associated with an uncontrollable desire to eat. ‘*Nervios*’ was described as a syndrome that includes somatic complaints (tremor, sweats, insomnia). According to Peruvian older adults, the causes of depression and anxiety included family and financial problems, loss of independence, loneliness, and past traumatic experiences. Ways of coping included self-reflection and adaptation to current situations, do your part, and obtaining emotional support from different sources (mainly relatives, friends, acquaintances, religion)

Emerging themes were organized into three main sections: (1) experience and perceptions of depression and anxiety; (2) causes of depression and anxiety; and (3) ways of coping with depression and anxiety (Fig. 1).

### Experience and perceptions of depression and anxiety

For most participants, the word ‘depression’ was more familiar than ‘anxiety.’ Older adults mentioned that people who are depressed express sadness, a reluctance to do things, and feel lonely, hopeless, and useless. Some older adults said that those feelings can be accompanied by headaches. One woman, who had seen a psychiatrist previously for depression treatment but had no current symptoms of depression/anxiety explained how she felt when she had depression:

*‘You do not feel like doing anything and everything seems monotonous, absurd, you do not see meaning in your life …everything, every day is the same’ (61* *year*-*old woman)*

Some older adults (7/39) felt such desperation that they mentioned suicidal ideation in their narratives.

*‘Last year, as I was crossing a bridge, it crossed my mind what it would like to toss myself over (laughs). Would I kill myself? I thought to myself. Now it scares me, I don’t go to that bridge, I don’t go up there like I used to’ (64* *year*-*old woman)*

On the other hand, when we asked, ‘How does a person with anxiety feel?’ or even ‘How do you feel when you are anxious?’ a considerable group (20/38) had no answer and replied *‘I don’t know.*’ Among those who replied, the most common response associated with the term ‘anxiety’ was having an uncontrollable desire to eat.

*‘That I understand well, that anxiety to eat, [like when] you’re hungry, you’ve had lunch, eaten everything, but you still want food, that’s anxiety.’ (60* *year*-*old woman)*

The idioms nerves (‘*nervios*’), sick from nerves (‘*enfermo de los nervios*’), and nervousness (*‘nerviosismo’*) were evoked in roughly half of older adults’ narratives, especially among the oldest old. Those idioms were associated with someone who is severely worried and experience tremor, restlessness, headaches, and sweating.

*‘I worried a lot, I have a son that rents his house, and what he earns is not enough to pay, all those things worried me. From anything, I would worry immediately. I am a nervous person, I suffer from nerves, my whole body begins to tremble.’ (Woman, 81* *year*-*old)*

Remarkably, two of older adults who had received or were taking anxiolytics mentioned that their physician prescribed them to treat their ‘*nervios.*’

‘*There is a pill that I take that relaxes me a little bit, a little pill that the doctor had given me. [I take them] in the mornings, to relax, to be calm during the day, because otherwise I am desperate, worried, thinking’ (81* *year*-*old woman).*

Although we asked about depression and anxiety separately, the vast majority of older adults (20/38) used the word ‘anxiety’ to express how they feel when they are ‘depressed’, and vice versa. Sometimes older adults also used the word ‘stress’ (‘*estresado’*) to define depression or anxiety, though a small group of participants (4/38) do mentioned the differences between having depression and anxiety.

*‘Depression is when you’re already depressed, so you don’t want to do anything. Anxiety is when you are there staring [blankly into space] without knowing what to do’ (69* *year*-*old man)*

### Causes of depression and anxiety

The principal causes of depression and anxiety expressed by Peruvian older adults followed four themes: ‘family and financial problems,’ ‘loneliness,’ ‘loss of independence’ and ‘past traumatic experiences.’

#### Family and financial problems

The most common cause of depression/anxiety mentioned was having ‘life problems,’ which usually referred to problems within the family (poor relationships and/or financial constraints). Older adults worried about their own financial situations and also about the situation of relatives. Moreover, most participants live with relatives in the same household (mainly children and extended family), and older persons are often present when disputes or arguments arise.

*‘You know, from so much worrying, so much fuss, and your child doesn’t have work, your husband, one worries, you know? So many problems, I think that’s what it comes from, depression’ (64* *year*-*old woman)*

#### Loneliness

Feeling lonely was described as a major cause of depression/anxiety in almost all older adults. It is important to note that here we refer to *feeling lonely* as opposed to *living alone*. Older adults indicated it is not enough to simply live in the same place as relatives, to have food or a roof over their heads. Older adults seek a genuine interest in them, their health, and wellbeing from relatives and others in their social network.

*‘I feel like alone, that’s why I have entered into a state of anxiety…, ‘you feel alone, [your children] never even ask how you are doing. Did you drink something? Did you eat? What did you do today? Nothing, you have children there just for decoration.’ (60* *year*-*old woman)*

Most participants stated that children have an inherent duty to take care of their parents, or at least to visit them. Several participants expressed that they worked hard for the sole benefit of their children, giving them as much as they could of themselves. When this inherent duty is not met, loneliness increased:

*‘When, for instance, a son that I have, I don’t even know how long it has been, [he] is an official in the air force. It has already been six months since he last visited me. Those things make you sad, because when you raise children, with love even if it’s under [the stresses of] poverty, they are still your children. Later, they forget about you…That [really] affected me, that’s how depression has affected me.’ (61* *year*-*old woman)*

#### Loss of independence

Few older adults directly linked depression or anxiety with having a chronic disease. Nevertheless, chronic disease was highlighted when associated with loss of independence to do activities they want to do. Sensory (auditory and visual) and mobility problems (e.g. chronic back or knee pain) were most distressful, especially among the oldest old. Sensory and mobility problems affect the ability to interact with friends and family members, making them embarrassed and self-conscious, reinforcing isolation from others and feelings of loneliness:

*‘[When] my son comes over, he talks with me, sometimes I can’t hear him (laughs), which makes me uncomfortable, and well, [my hearing problem] is never going to be fixed. I tell my wife about it, she tells me not to worry… but it bothers me’ (80* *year*-*old man)*

*‘Sometimes I start to think about it, I think my destiny is to live alone, you know? Here, under this roof, … the bad thing is that I can’t see,…the cataract messed up my vision… If my vision were ok, I would leave [the house], I would sit outside, or I would go out to the soccer field, or be able to see when someone is passing by..’ (88* *year*-*old man)*

#### Past traumatic experiences

Most of the older adults interviewed (30/38) had vivid memories of traumatic episodes during childhood that they continue to think about in older age, especially women, who mostly reported trauma related to sexual violence. Some men also reported trauma related to work exploitation when they were children or teenagers. We included traumatic experiences as a cause of depression because older adults seem to have unresolved thoughts about those situations that appeared to contribute to increased feelings of loneliness, depression, and anxiety. The following woman almost at the beginning of the interview shared why she thinks she had depressive symptoms:

*‘The thing is that this sadness, this feeling, this anger I have had since I was a girl. My mom was not a good person…she made one of those shamans [sexually] abuse me. Ever since, this [experience] has made it impossible for me to be happy. I thought to myself…so many times I asked myself (cries), how could she be my mother and do that to me?’ (63* *year*-*old woman)*

### Ways of coping with depression and anxiety symptoms

We asked participants what they do to reduce feelings of depression and anxiety. As family problems, loneliness, loss of independence, and past trauma co-exist and interact, the overall coping strategies were to stop thinking too much and to avoid constantly ruminating on their problems. We summarize older adults’ strategies under four rubrics: ‘Self-reflection and adaptation,’ ‘do your part”, and ‘get emotional support’ from different sources.

#### Self-reflection and adaptation

Some older people mentioned that it is important to self-reflect and accept that some situations may be beyond your control. One older woman summarized this idea by saying that ‘*we can’t carry the weight of the whole world’;* rather, one must accept certain aspects of life.

*‘I didn’t want to live, but I said ‘No, why do I have to be like that? Yes, my daughter has left. Everyone has to leave. [But] no, my children are not my property.’ (70* *year*-*old man)*

*Do your part (“poner de tu parte”*)

Older adults highlighted that people have to *do their part* as a key step in recovery, to make an effort to think positively. It was a common idea that nobody will recover from having depression/anxiety if a person does not make an effort:

*‘To feel better, I have to think about cheering myself up …who can cheer me up? Nobody…I have to just [do it myself], my problem has to be carried by myself alone’ (75* *year*-*old man)*

Older people expressed that they often try to distract their minds to avoid thinking too much. People described different ways of distracting themselves such as going out, continuing to work (if one enjoys work), or even doing activities at home (e.g. knitting, watching TV, or listening to the radio).

*‘Because (working) is my therapy, yes! Thank God that I like what I do, I love weaving, and I thank the woman who appeared on my path, who taught me and started me in this work, in this trade that I’m very grateful for’ (62* *year*-*old woman)*

#### Get emotional support

Older adults talked about seeking emotional support from different sources, such as in religion/God, family members, acquaintances or people from the community, and health professionals. They mentioned that support (*‘apoyo’*) is fundamental to recovery. Most participants (35/39) mentioned faith in God as a source of emotional support. Older adults stated that having the support of God relieved them from their problems, made them stronger, and gave them peace. Furthermore, some older adults stated that they do not need psychologists to reduce their symptoms because they have God.

*‘My psychologist is God, and I prayed to him every night to take those [feelings] away from me, and little by little, they went away.’ (61* *year*-*old woman)*

Some older adults (15/38) said that relatives should help someone distract themselves, do activities together, or cheer them up. Likewise, older adults stated that having close, trustworthy friends helps reduce loneliness and depressive symptoms. A group of older adults (12/38) mentioned that sometimes it is easier and more comfortable to have a conversation with someone other than a relative or close friend. Therefore, some older adults sought out strangers or acquaintances for conversation, as they might give them a more ‘objective’ point of view regarding their problems. A taxi driver explained:

*‘What I do sometimes is, since I’m a taxi driver, and someone [mature] and prepared [gets in my taxi] and gives me an opportunity to tell them my problems, I feel relieved, because if not, it stays inside of me. You don’t have anyone, if you tell your family, they will tell someone else, your brother, brother*-*in*-*law, and it becomes [gestures “something bigger” with hands], but people that don’t know you, you’ll never see again.’ (63* *year*-*old man)*

Some older adults described participating in social groups such as Seniors Clubs (‘Club del Adulto Mayor’), which are composed of peers engaging in leisure activities or participating in religious groups where people can pray together. Older adults in such social groups expressed a sense of support from the group, which seemed to help older adults to stop dwelling on their problems.

*‘I like [the Seniors Club], mostly because, as you see here, I am by myself, and at the [Seniors Club], at least, I laugh, I play’ (72* *year old woman)*

Very few older adults mentioned psychologists as sources of support or relief. Psychiatrists were even less commonly mentioned. Older adults usually reported that they did not share feelings with a physician because ‘*[the physicians] do not have time*.’ Older adults who had appointments with a mental health professional did not go directly to them but were referred after being seen for a different condition such as heart problems or headaches. Some who had had appointments with a psychologist reported helpful experiences, and some reported ‘*it was a waste of time’* because the visit consisted of just talking or revisiting negative experiences.

## Discussion

Our study explored the lived experiences of depression and anxiety among Peruvian older adults from low-resource settings. The ideas, concepts, causes, and coping strategies for depression and anxiety overlap. Depression was represented mainly as sadness, loneliness, and lack of desire to do things. The word ‘anxiety,’ in turn, was difficult to define, with the word being associated, in several cases, with a compulsive desire to eat. ‘*Nervios,’* and ‘*enfermo de los nervios*’ were idioms used to describe a worrying state that generates tremor, insomnia, and restlessness. Peruvian older adults indicated that the main causes of depression and anxiety symptoms are constant familial and economic problems, loneliness, loss of independence and past traumatic experiences (childhood). Coping strategies Peruvian older adults use to reduce depression and anxiety symptoms include self-reflection and adaptation, ‘do your part’ (*poner de tu parte*), and getting emotional support mainly from non-professionals such as relatives, friends, acquaintances, and God/religion.

Peruvian older adults described being depressed by emphasizing emotional symptoms such as sadness, loss of interest, and loneliness, which is consistent with other qualitative studies [17, 28]. On the other hand, older adults associated anxiety with compulsive eating, which highlights the feeling of loss of control [29]. Some studies have hypothesized that individuals with anxiety symptoms cope by using momentary experiences of pleasure, such as eating, to forget real-life problems [30]. Moreover, in the Peruvian sample, while the word anxiety did not trigger answers related to general anxiety disorders (GAD), idioms such as nerves (*‘nervios’)* did describe a state that includes characteristics of GAD such as restlessness and tremors [31]. *‘Nervios’* has been frequently described among Mexicans and other Hispanic countries [28, 32]. The study of *‘nervios’* is extensive, and it has been suggested that it is a syndrome of chronic dysphoric mood with somatic complaints [33] whose origins are rooted in social problems [34]. Independently of the exact definitions, for some Peruvians older adults, *‘nervios’* were perceived as something that can be mitigated with anxiolytics. Considering the frequency and characteristics of use, *‘nervios’* may be a gateway for exploring symptoms of anxiety in Peruvian and other older Latino populations.

Peruvian older adults described loneliness as a feature of depression and also as precursor for it, which is compatible with several studies of depression in older adults [22, 35]. Loneliness has independent detrimental effects on cognition, mobility, and daily life activities [36, 37]. Older adults do not require simply the presence of others (in fact, most Peruvian in our study live with their relatives), but also the presence of individuals with whom they can trust, interact, and work together [38]. From the point of view of Peruvian older adults, children often do not carry out their inherent duty of taking care of them. Although it is unknown if older adults fostered good relationships with their offspring when they were children, traditional values in Latin America such as family closeness seem difficult to achieve for younger generations, as they often have competing obligations and expectations.

Regarding the perceived link between chronic diseases and depression or anxiety, qualitative studies among older adults from different settings have reported this link [17, 39]. Nevertheless, in our sample, what caused stress in Peruvian older adults was not the diseases themselves but the impact on their ability to interact with people and/or to continue doing what they want. This idea is concordant with the WHO patient-centered care emphasis on functional ability and maintenance of independence, rather than multi-morbidity, in older adults [40, 41].

Peruvian older adults stressed the importance of prior traumatic experiences, which are perceived as the origin of depression/anxiety symptoms. We believe that experiencing trauma was common because the majority of our participants came from socially disadvantaged backgrounds. Similarly, a study that explored the experiences of disadvantaged African American women found that women associated the origin of their depression to their history of trauma, including sexual abuse and poverty [42].

Regarding strategies to cope with depression and anxiety in our study, older adults often stated that reducing depressive and anxiety symptoms comes from within themselves. The main ideas was ‘self-reflection and adaptation’ to circumstances you cannot change and ‘doing your part,’ making an effort to feel better. Several studies among older adults have highlighted the importance of oneself in overcoming depression, calling it ‘inner strength’ or ‘self-reliance’ [16, 43, 44]. Viewing personal responsibility as key to overcoming depression and anxiety can be seen in two opposing ways: as a potential barrier to health seeking behavior, since someone who sees the solution in their own mind may not recognize the purpose of seeking care from a professional [23], or as a potential gateway to introducing psychological techniques to strengthen cognitive self-coping mechanisms [43].

Coping strategies also included seeking emotional support through interacting with someone he/she trusts. In our study, several participants expressed family problems and were ashamed to disclose their thoughts with relatives because they did not think they would be understood. Similarly, studies in European samples mentioned that older adults did not want to burden relatives with more problems [45], while in a group of Korean-American individuals, it was considered a sign of weakness to share problems with one’s social network [23]. In general, our findings suggest that Peruvian older adults are willing to receive support from sources outside their inner family circle. Additionally, religion and God was mentioned as an important way of alleviating negative feelings. Activities such as prayer, talking to a pastor, or going to church were cited as ways to obtain mental strength and reduce depression/anxiety symptoms. Other studies has described an increasing capability to manage difficult situations in individuals with a spiritual relationship with God [46].

The strengths of this study include our leveraging a large project that measured quantitative scores of depression and anxiety, which allowed us to purposively select older adults with a wide range of illness experience of depression and anxiety. In addition, we carried out interviews and data analysis in the native language of the participants, which enabled us to more deeply understand the situations, analogies, experiences and idioms used. Regarding limitations, first, since the parent study excluded older adults that were physically unable to perform spirometry, we may have missed highly dependent older individuals with different illness experiences. Second, since most of our sample were ‘younger old,’ our results could reflect an illness experience closer to the perspectives of younger adults. Nonetheless, we did include seven participants that belonged to the ‘oldest old’ category (≥ 75 year-old). Finally, the sample was composed mainly of low-income Peruvian older adults living in semi-urban areas. Older adults from rural areas or that have better socioeconomic status might have different views of depression and anxiety.

This study has implications for community interventions in older adults. First, mental health initiatives should include common expressions and idioms such as ‘*nervios*’ that older adults used because it is a gateway to talking about disturbing feelings and somatic symptoms. Second, any intervention that attempts to improve mental health in older adults should address loneliness. Public health researchers and practitioners should treat loneliness as a health problem, which means active screening and provision of effective social and psychological interventions [37]. Third, while a comprehensive approach for multi-morbidity is important, special attention should be given to conditions that reduce the capacity of older adults to engage in social interactions. For instance, hearing aids could have a tremendous positive impact for older adults’ daily lives. Fourth, interventions for older adults from underserved areas should systematically screen for childhood traumatic experiences. Attachment-based psychological interventions appear beneficial for individuals with adverse experiences [47]. Fifth, personal responsibility to cope with depression and anxiety is a natural coping mechanism. Instead of discouraging it, it may be an opportunity to introduce psychological techniques that can be used for older adults to help themselves. Finally, Peruvian older adults were receptive to obtaining emotional support from non-professionals. Community interventions might incorporate individuals and social groups, working in tandem with the health system, trained to provide mental health care or act as navigators between older adults and health professionals.

## Conclusions

Understanding and taking into account the illness experiences of older individuals lay the foundations for the design of feasible and acceptable community-based mental health interventions tailored to the needs and contexts of older adults.

## Supplementary information

**Additional file 1.** Interview guide in Spanish and English.

## Data Availability

The data used or analysed during the current study are available from the corresponding author on reasonable request.
