# Correction to: “We can’t carry the weight of the whole world”: Illness experiences among Peruvian older adults with symptoms of depression and anxiety

**DOI:** 10.1186/s13033-020-00403-5

**Published:** 2020-09-17

**Authors:** Oscar Flores-Flores, Alejandro Zevallos-Morales, Ivonne Carrión, Dalia Pawer, Lorena Rey, William Checkley, John R. Hurst, Trishul Siddharthan, Jose F. Parodi, Joseph J. Gallo, Suzanne L. Pollard

**Affiliations:** 1 Facultad de Medicina Humana, Centro de Investigación del Envejecimiento (CIEN), Universidad de San Martin de Porres, Lima, Peru; 2 Asociación Benéfica PRISMA, Lima, Peru; 3 Facultad de Medicina Humana, Universidad de San Martin de Porres, Lima, Peru; 4 Pontificia Universidad Católica del Perú, Lima, Peru; 5 Division of Pulmonary and Critical Care, School of Medicine, Johns Hopkins University, Baltimore, MD USA; 6 Department of International Health, Bloomberg School of Public Health, Johns Hopkins University, Baltimore, MD USA; 7 Center for Global Non-Communicable Disease Research and Training, School of Medicine, Johns Hopkins University, Baltimore, MD USA; 8 UCL Respiratory, University College London, London, UK; 9 Department of Mental Health, Bloomberg School of Public Health, Baltimore, MD USA; 10 Department of General Internal Medicine, School of Medicine, Johns Hopkins University, Baltimore, MD USA

## Correction to: Int J Ment Health Syst (2020) 14:49 10.1186/s13033-020-00381-8

Unfortunately, in the original publication of the article, the sixth, seventh and eighth authors’ first names in the author group were published in abbreviated form as “W. Checkley, J. R. Hurst, T. Siddharthan”. The first name of the authors should be spelled out and should appear in full form as “William Checkley, John R. Hurst, Trishul Siddharthan”.
