# Supplementary material for: “We can’t carry the weight of the whole world”: illness experiences among Peruvian older adults with symptoms of depression and anxiety
Source: Int J Ment Health Syst. 2020 Jul 10;14:49. doi: 10.1186/s13033-020-00381-8 (PMC7350592; doi:10.1186/s13033-020-00381-8)
Supplement: Supplementary file 1 — Additional file 1. Interview guide in Spanish and English. [file 13033_2020_381_MOESM1_ESM.docx]

[SPANISH]

**Guía de Entrevista**

**INTRODUCTION**

Antes de comenzar,

¿Dónde nació?

¿Cuánto tiempo ha vivido en San Juan de Miraflores?

¿Con quién(es) vive?

¿En qué trabaja o trabajaba?

**Parte 1**

En esta parte le voy a leer una historia. Luego, le pregunto qué opina sobre ella:

**Caso 1**

**Si es Mujer**

La **Sra. R** es una mujer de (edad de R) que era profundamente religiosa y era muy activa en su iglesia. Ella estaba convencida que Dios no le iba a poner pruebas que ella no podría soportar.

Ella tomaba pastillas para el corazón tres veces al día e inyecciones de insulina cada mañana. Generalmente la **Sra. R** manejaba las cosas bien, pero recientemente le dijo a su pastor que se sentía "*abrumada por el mundo*". Se dio cuenta que no disfrutaba de su comida y que perdía peso. Otros notaron que ella estaba muy olvidadiza y que ya no se arreglaba el cabello ni se maquillaba. El médico la examinó e hizo varias pruebas de sangre y le dijo que su salud no había cambiado. Sin embargo, la **Sra. R** no se sentía bien.

1. ¿Cuál piensas que es el problema de la Sra. R?
2. ¿Qué se debería hacer al respecto?
3. ¿Quién podría ayudarla?

**Si es hombre**

El Sr. **R** es una hombre de (edad de R) que era profundamente religioso y era muy activo en su iglesia. Él estaba convencido que Dios no le iba a poner pruebas que ella no podría soportar.

Él tomaba pastillas para el corazón tres veces al día e inyecciones de insulina cada mañana. Generalmente el **Sr. R** manejaba las cosas bien, pero recientemente le dijo a su pastor que se sentía "*abrumado por el mundo*". Se dio cuenta que no disfrutaba de su comida y que perdía peso. Otros notaron que estaba muy olvidadizo y que ya no le importaba su apariencia. El médico lo examinó e hizo varias pruebas y le dijo que su salud no había cambiado. Sin embargo, el **Sr. R** no se sentía bien.

1. ¿Cuál piensas que es el problema del Sr. R?
2. ¿Qué se debería hacer al respecto?
3. ¿Quién podría ayudarlo?

**Caso 2**

**SI ES MUJER**

La Sra. B es una mujer de R (edad de R) cuyo esposo murió hace 8 años. Junto a su esposo criaron a tres hijos que ahora están casados, tienen sus propias familias y viven en diferentes departamentos. Ella recibe visitas de sus hijos una vez al año. Ella solía reunirse con sus amigas todos los miércoles. Sin embargo, últimamente apenas puede salir de la cama por la mañana, mucho menos vestirse y salir con sus amigas. Ella no tiene energía y ha perdido 4 kilos. El médico la examinó e hizo varias pruebas y le dijo que su salud no había cambiado. Sin embargo, la señora B no se sentía bien.

1. ¿Cuál piensas que es el problema de la Sra. R?

2. ¿Qué se debería hacer al respecto?

3. ¿Quién podría ayudarla?

**SI ES HOMBRE**

El Sr. B es un hombre de R (edad de R) cuya esposa murió hace 8 años. Junto a ella, criaron a tres hijos que ahora están casados, tienen sus propias familias y viven en diferentes departamentos. Él recibe visitas de sus hijos una vez al año. Él solía reunirse con sus amigos todos los miércoles. Sin embargo, últimamente, apenas puede salir de la cama por las mañanas, mucho menos vestirse y salir con sus amigos. Él no tiene energía y ha perdido 4 kilos. El médico lo examinó e hizo varias pruebas y le dijo que su salud no había cambiado. Sin embargo, el Sr. B no se sentía bien.

1. ¿Cuál piensa que es el problema de la Sra. R?

2. ¿**Qué** se debería hacer al respecto?

3. ¿**Quién** podría ayudarla?

**Opcional**

El Sr. M es un barbero de 71 años que tiene un dolor constante en su espalda y rodillas. Últimamente, no tiene interés en ver sus programas favoritos en la televisión. Su esposa está frustrada porque su esposo ha dejado de ir a la iglesia y le parece que está muy olvidadizo. Además, se despierta en las noches y no puede volver a quedarse dormido. Se siente exhausto y con menos energía. Un doctor lo examinó cuidadosamente y no encontró algún cambio en su salud. Sin embargo, el Sr. M no se sentía bien.

1. ¿Cuál piensa que es el problema del Sr. M?

2. ¿**Qué** se debería hacer al respecto?

3. ¿**Quién** podría ayudarla?

**Parte 2.**

En esta parte le preguntaré sobre su salud física y mental. Me gustaría que use sus propias palabras.

**Entender la percepción sobre depresión**

- 1. Alguna vez, ¿se ha considerado como alguien que podría estar **con depresión**?

¿Cómo se siente Ud. cuando está con depresión (**deprimido)**?

Si la respuesta es NO - *¿Alguna vez, se ha considerado como alguien* ***que está triste la mayor parte del tiempo***?)

Si la respuesta es NO - ¿Alguna vez has conocido a alguien con depresión, cómo es?

*(Luego, ¿Esa descripción se parece a cómo se siente Ud.?*)

¿Cómo son las personas con **depresión**?

- 1. ¿Cuándo esos sentimientos empezaron? (temporalidad)
  2. ¿Por qué cree que estos sentimientos empezaron? (causalidad)
  3. ¿Cómo la depresión interfiere con su día? ¿Cómo interfiere con su noche?
  4. ¿De qué manera la depresión afectó/afecta sus relaciones con su familia?
  5. ¿De qué manera la depresión afectó/afecta sus relaciones con sus amigos?
  6. ¿De qué manera la depresión afecta /afectó sus relaciones con otras personas?
  7. ¿Cómo crees que la depresión afecta tu salud?

- 1. ¿Qué hace para sentirse mejor cuando se siente deprimido?
  2. La última vez que fue al médico, ¿por qué fue?

1. ¿Le contó al médico sobre sus problemas de sentirse deprimido o triste?
   1. Si es No - ¿Por qué no le contó?
   2. Si es Sí - ¿Qué le dijo el Doctor?
   3. Respecto a **medicinas o medicamentos.** ¿Ud. considera que medicinas pueden ayudar a las personas con depresión?

(Si es Sí - ¿habrá personas que no les funcioné? ¿A quiénes les podría funcionar?

- 1. Algunas personas mencionan que *hablar sobre las cosas* les ayuda cuando están deprimidas. ¿Qué piensas sobre la **terapia o consejería psicológica**?
  2. ¿Dónde las personas pueden aprender acerca de la depresión?

**Entender percepción de Ansiedad**

- 1. ¿Alguna vez se ha considerado como alguien con ansiedad?

¿Cómo se siente Ud. cuando está ansioso/a? ¿Conoce Ud. alguien que sufra de ansiedad?

¿Durante el día o la noche, se siente muy preocupada o intranquila o muy nerviosa?

Respuesta es SI ¿Qué cosas/situaciones le genera esas sensaciones?

¿Por qué cree que estos sentimientos empezaron?

- 1. ¿Ud. considera que es posible que una persona tenga depresión y también ansiedad?

**Relación con otras Enfermedades Crónicas**

- 1. ¿Qué problemas de salud tiene que afecten su estado de ánimo o la forma en que se siente emocionalmente?

**Si reporta una enfermedad física o problema, “Cuénteme cómo le afecta esa condición?**

- 1. ¿Estas tomando alguna medicina? (Explorar un poco sobre sus medicamentos)
  2. Qué hay de lo contrario, ¿su estado de ánimo o sus emociones afectan la forma en que se siente físicamente? ¿En qué manera?
  3. ¿Cómo crees que te sentirás en 1 año? ¿En 3 años? (especialmente respecto a su salud mental)

**Barreras y facilitadores para acceder a un cuidado de salud mental profesional**

- 1. Desde su punto de vista, ¿Cuáles son los obstáculos que las personas con depresión (o ansiedad) tienen para recibir ayuda profesional?
  2. ¿Qué permite o ayuda a que personas con depresión(o ansiedad) reciban ayuda profesional?
  3. En caso quisieras recibir ayuda del centro de salud de tu comunidad para la depresión/ansiedad, ¿qué problemas crees que tendrías que afrontar?
  4. Con respecto a profesionales en salud ¿Cuáles es la experiencia que usted ha tenido con en estos profesionales? (Especialmente los que han tenido experiencia con profesionales de salud mental)

**Finales**:

¿Alguna cosa sobre depresión o ansiedad que Ud. Quisiera contarme?

¿Tendría alguna recomendación para nosotros, de cómo ayudar a las personas con depresión y ansiedad?

¿Alguna pregunta para nosotros?

Gracias por su tiempo.

[ENGLISH]

**Introduction**

Before starting,

Where were you born?

How long have you lived in San Juan de Miraflores?

Who do you live with?

What is/was your job?

**Part 1**

**Case 1**

**MALE SCENARIO:**

Mr. B is a (R’s AGE) year old man whose wife died 8 years ago. They raised three children who are now all married with families of their own and living in different states. He has good visits with them about once a year. He used to play cards with friends every Wednesday. Lately, though, he can hardly drag himself out of bed in the morning, let alone get himself dressed and out to a card game. He has no energy and lost 12 pounds. His doctor examined him thoroughly and found his health to be the same. Nevertheless,

Mr. B does not feel well.

**FEMALE SCENARIO:**

Mrs. B is a (R’s AGE) year old woman whose husband died 8 years ago. They raised three children who are now all married with families of their own and living in different states. She has good visits with them about once a year. She used to play cards with friends every Wednesday. Lately, though, she can hardly drag herself out of bed in the morning, let alone get herself dressed and out to a card game. She has no energy and lost 12 pounds. Her doctor examined her thoroughly and found her health to be the same.

Nevertheless, Mrs. B does not feel well.

A. What do you think is the matter with (Mr./Mrs.) B?

B. What should be done about it?

C. Who could help?

**Case 2**

**MALE SCENARIO:**

Mr. B is a (R’s AGE) year old man whose wife died 8 years ago. They raised three children who are now all married with families of their own and living in different states. He has good visits with them about once a year. He used to play cards with friends every Wednesday. Lately, though, he can hardly drag himself out of bed in the morning, let alone get himself dressed and out to a card game. He has no energy and lost 12 pounds. His doctor examined him thoroughly and found his health to be the same. Nevertheless,

Mr. B does not feel well.

**FEMALE SCENARIO:**

Mrs. B is a (R’s AGE) year old woman whose husband died 8 years ago. They raised three children who are now all married with families of their own and living in different states. She has good visits with them about once a year. She used to play cards with friends every Wednesday. Lately, though, she can hardly drag herself out of bed in the morning, let alone get herself dressed and out to a card game. She has no energy and lost 12 pounds. Her doctor examined her thoroughly and found her health to be the same. Nevertheless, Mrs. B does not feel well.

A. What do you think is the matter with (Mr./Mrs.) B?

B. What should be done about it?

C. Who could help?

**Part 2**

In this part I will ask about your physical and mental health. I would like you to use your own words.

**Understand the perception of depression**

3.1 Have you ever considered yourself as someone who could be with depression?

How do you feel when you are depressed (depressed)?

If the answer is NO - Have you ever considered yourself as someone who is sad most of the time?)

If the answer is NO - Have you ever met someone with depression, how is it?

(Then, does that description resemble how you feel?)

How are people with depression?

3.2 When did those feelings start? (temporality)

3.3 Why do you think these feelings started? (causality)

3.4 How does depression interfere with your day? How does it interfere with your night?

3.5 How did depression affect / affect your relationships with your family?

3.6 How did depression affect / affect your relationships with your friends?

3.7 How does depression affect / affect your relationships with other people?

3.8 How do you think depression affects your health?

3.9 What do you do to feel better when you feel depressed?

3.10 Last time you went to the doctor, why was it?

Did you tell the doctor about your problems feeling depressed or sad?

If it is No - Why didn't you tell him?

If Yes - What did the Doctor tell you?

3.11 Regarding medicines. Do you think medicines can help people with depression?

(If yes - will there be people who didn't work for them? Who could it work for?

3.12 Some people mention that talking about things helps them when they are depressed. What do you think about psychological counseling or therapy?

3.13 Where can people learn about depression?

**Understand anxiety perception**

3.14 Have you ever considered yourself as someone with anxiety?

How do you feel when you are anxious? Do you know someone who suffers from anxiety?

During the day or night, do you feel very worried or restless or very nervous?

If answer is **YES** What things / situations generate those feelings?

Why do you think these feelings started? When?

3.15 Do you Do you think it is possible for a person to have depression and also anxiety?

**Relationship with other Chronic Diseases**

3.16 What health problems do you have that affect your mood or the way you feel emotionally?

If you report a physical illness or problem, “Tell me how that condition affects you?

3.17 Are you taking any medicine? (Explore a little about your medications)

3.18 What is the opposite, does your mood or emotions affect the way you feel physically? Which way?

3.19 How do you think you will feel in 1 year? In 3 years? (Especially regarding your mental health)

**Barriers and facilitators to access professional mental health care**

3.20 From your point of view, what are the obstacles that people with depression (or anxiety) have to get professional help?

- 1. What allows or helps people with depression (or anxiety) receive professional help?

3.22 If you would like to receive help from your community health center for depression / anxiety, what problems do you think you would have to face?

3.23 Regarding health professionals, what experience have you had with these professionals? (Especially those who have had experience with mental health professionals)

**Finals:**

Anything about depression or anxiety that you would like to tell me?

Would you have any recommendations for us, how to help people with depression and anxiety?

Any questions for us?

Thanks for your time.
